# Five-year follow-up of corneal endothelial cell density after transscleral ab interno glaucoma gel stent implantation

**DOI:** 10.1007/s00417-022-05898-x

**Published:** 2022-11-25

**Authors:** Markus Lenzhofer, Armin Motaabbed, Hans Peter Colvin, Melchior Hohensinn, Veit Steiner, Wolfgang Hitzl, Christian Runge, Sarah Moussa, Herbert A. Reitsamer

**Affiliations:** 1 Department of Ophthalmology and Optometry, Paracelsus Medical University Salzburg, Salzburger Landeskliniken, Muellner Hauptstrasse 48, 5020 Salzburg, Austria; 2 Research Program Experimental Ophthalmology, Paracelsus Medical University Salzburg, Salzburger Landeskliniken, Muellner Hauptstrasse 48, 5020, Salzburg, Austria

**Keywords:** XEN, XEN Glaucoma Gel Microstent, MIGS, Endothelial cell count, Endothelial cell density

## Abstract

**Purpose:**

This study investigates the course of the endothelial cell density over a period of 5 years after XEN45 implantation (XEN45µm, Allergan Plc., USA) with or without combined cataract surgery.

**Methods:**

This is a prospective, cross-sectional, monocentric, non-randomized clinical trial with the intention to treat a population of the University Eye Clinic Glaucoma Service Salzburg. One hundred and fifty-five eyes with preoperative central corneal endothelial cell counts were subjected to XEN45 implantation with (combined surgery group) or without (solo surgery group) combined cataract surgery. Endothelial cell density was measured at 3 corneal positions. XEN45 location parameters were determined with anterior segment OCT and gonioscopy.

**Results:**

In the combined surgery group, a significant reduction of central endothelial cell count was found at years 2 and 4 when compared to baseline (*p* = 0.001 and *p* = 0.02, *n* = 86), whereas at years 1, 3, and 5, no change was detected (all *p* > 0.09). The median reduction of endothelial cell count was − 79 (95% CI: − 183 to − 9) and − 93 (95% CI: − 220 to 23) cells at years 2 and 4, respectively. In the solo surgery group (*n* = 69), no significant change in endothelial cell counts was detected at any time during the 5-year evaluation period (all *p* > 0.07). Explorative data analyses revealed that XEN45 location parameters did not significantly influence the course of endothelial cell count over time.

**Conclusions:**

Endothelial cell loss after XEN45 implantation seems to be low. The present data suggest no impact on the position of the implant with regard to central endothelial cell counts in this study.

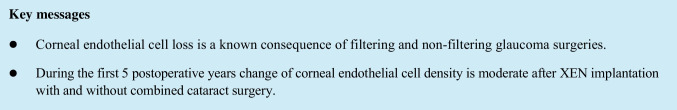



## Introduction

Glaucoma is a leading cause of irreversible visual function loss worldwide [1]. Although being a multi-factorial disease with many known risk factors, the current state-of-the-art therapy is the reduction of intraocular pressure (IOP) to control disease progression. Most glaucoma patients are treated with IOP-lowering eye drops or laser treatments; however, an increasing number of patients require surgical interventions for IOP reduction [2–6].

All commonly performed filtering glaucoma surgeries are capable of lowering the IOP. Over the last decade, several new minimally invasive glaucoma surgeries have been introduced [7–9]. The XEN45 Glaucoma Gel Microstent (XEN 45 µm, Allergan Plc., USA) is a member of this new group of implants (MIGS, MIGS +) [10]. Like other surgical techniques, it bypasses aqueous humor from the anterior chamber to the subconjunctival space in order to lower IOP [11].

Corneal endothelial cell loss is a known consequence of filtering and non-filtering glaucoma surgeries, and it can reach up to − 18.6%, 2 years after surgery [12–16]. Chronic decrease in endothelial cell density can be detrimental to corneal fluid homeostasis. Corneal edema is the result of imbalanced fluid homeostasis of the cornea, which affects the vision of the patients.

The scientific literature provides limited data on the effect of XEN45 procedures on corneal endothelial cell survival. Hence, the present longitudinal study aims to investigate the change in endothelial cell count in patients after XEN45 solo surgery (solo surgery group) and patients after combined XEN45/cataract surgeries (combined surgery group) over an evaluation period of 5 years.

## Methods

This prospective, cross-sectional, non-randomized, monocentric clinical evaluation in a patient to treat population was conducted in a tertiary health care center. The study and data collection were carried out with approval from the appropriate institutional review board. The authors confirm that the study and data collection were conducted in conformity with all federal and state laws, informed consent was obtained, and the study was in adherence to the tenets of the Declaration of Helsinki. Clinical trial registration information is publicly available at www.clinicaltrials.gov. Hypotheses were defined prior study start.

The study cohort consists of two groups of intention to treat patients. One hundred and fifty-five consecutive eyes received either XEN45 implants only (solo surgery group) or combined surgery/cataract surgeries were performed (combined surgery group). Only patients with insufficiently controlled IOP or intolerance to topical therapy in mild to moderate open-angle glaucoma were included in the study. The feasibility to measure endothelial cell count was mandatory. Patients with further additional IOP-lowering procedures (except needlings, bleb revisions, YAG lasers, or Argon lasers) were excluded from the analysis. Surgeries took place between 2014 and 2019. All endothelial cell counts were recorded within 3 months preoperatively at baseline visit. All 155 eyes underwent 2 study visits: a baseline visit preoperatively between the years 2014 and 2019, and a last postoperative visit in the year 2021. Patients could choose to have corneal endothelial cell follow-ups yearly in between at the study center, but this was not obligatory. Of the eyes, 37% (58/155) used the offer of interim visits a minimum of once. As far as available, interim data between the XEN45 surgery date and the date of the last postoperative visit in 2021 were also included in the data analysis.

The primary endpoint was the change in central endothelial cell density at the last postoperative visit in comparison to preoperative data.

XEN45 surgeries were performed in accordance with our previously published XEN45 treatment algorithm [17]. Topical medications were changed to preservative-free eye drops for the duration of a minimum of 1 month preoperatively. Topical preservative-free corticosteroids (dexamethasone, TID^1^) were started additionally 2 weeks before surgery.

XEN45 gel stents were implanted ab interno after intra-tenon application of mitomycin C (0.05–0.2 ml, 4–16 μg MMC total).

In the solo surgery group, XEN45 was implanted in the superior-nasal quadrant via a 1.1-mm clear corneal inferior-temporal incision while stabilizing the eye with a second instrument (Phaco Spatula) via a 0.7-mm clear corneal superior-temporal incision. Cohesive viscoelastic (Healon GV) was used to maintain the anterior chamber depth during implantation. Gonioscopy was used intraoperatively while entering the sclera at the angle. The entry position of the XEN45 into the sclera was aimed anterior adjacent to Schlemm’s canal; the aimed length of the scleral tunnel was 2 mm with an exit point 3 mm posterior to the limbus. The remaining length of the XEN45 in the anterior chamber was adjusted to approximately 1 mm at the end of surgery. After removing the viscoelastic from the AC, an intracameral antibiotic was administered at the end of surgery (cefuroxime 0.1 ml).

In the combined surgery group, clear corneal ports from cataract surgery were used for subsequent XEN45 implantation. A temporal approach was used for all cataract surgeries. The same phacoemulsification machine was used in all eyes (Oertli Faros, Oertli, Switzerland). For cataract surgery, a dispersive viscoelastic was used in all eyes (MEDIO-CLEAR 2.0%, Aivimed, Germany). Subsequent XEN45 implantation was performed as described above.

Postoperatively, the patients received preservative-free topical corticosteroids for a minimum of 6 weeks (dexamethasone, minimum TID, first week Q1H^2^ during daylight) and preservative-free topical antibiotics for 1 week (ofloxacin, TID).

Two patient groups were analyzed separately and overall: the solo surgery group and the combined surgery group.

**Fig. 1:**
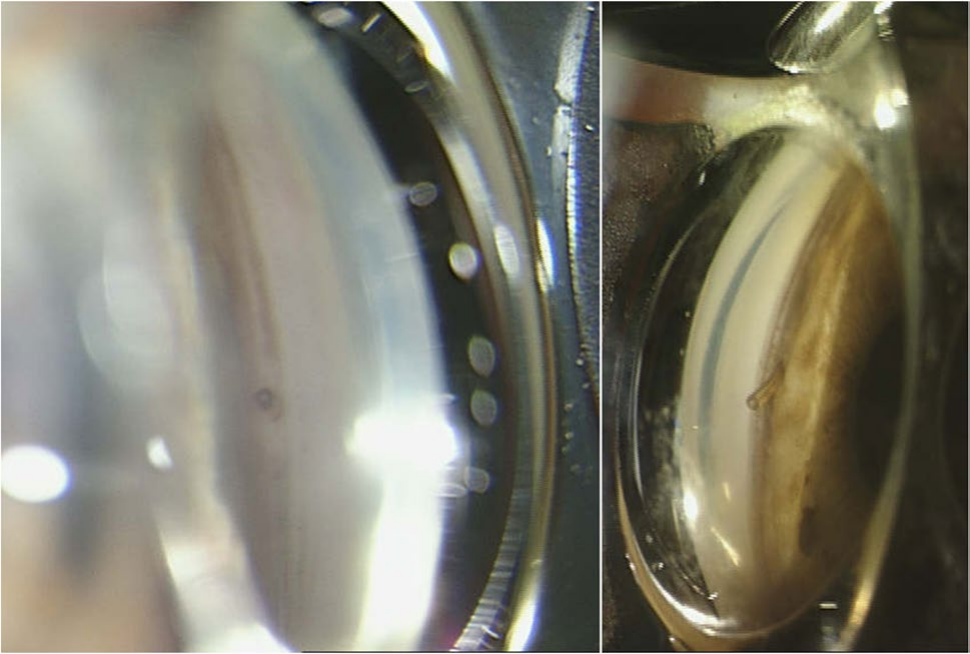
Gonioscopy and assessment of the XEN45 entry in the sclera. In these examples, the XEN45 enters the sclera at the pigmented trabecular meshwork (left) and at the scleral spur (right)

During their last visit in 2021, patients were assessed as to endothelial cell density and the position of the XEN45 measured by anterior segment optical coherence tomography and gonioscopy. Gonioscopy and angle by Shaffer Grade were assessed in the quadrant of XEN45 implantation (superior-nasal). The position of the XEN45 was graded as follows: anterior to Schwalbe line, in Schwalbe line, anterior to Schlemm’s Canal, in Schlemm’s Canal, in the non-pigmented trabecular meshwork, in pigmented trabecular meshwork, posterior to pigmented trabecular meshwork, at scleral Spur (see Fig. 1). The presence of a possible endothelial/iris touch of the XEN45 stent was also noted.

**Fig. 2:**
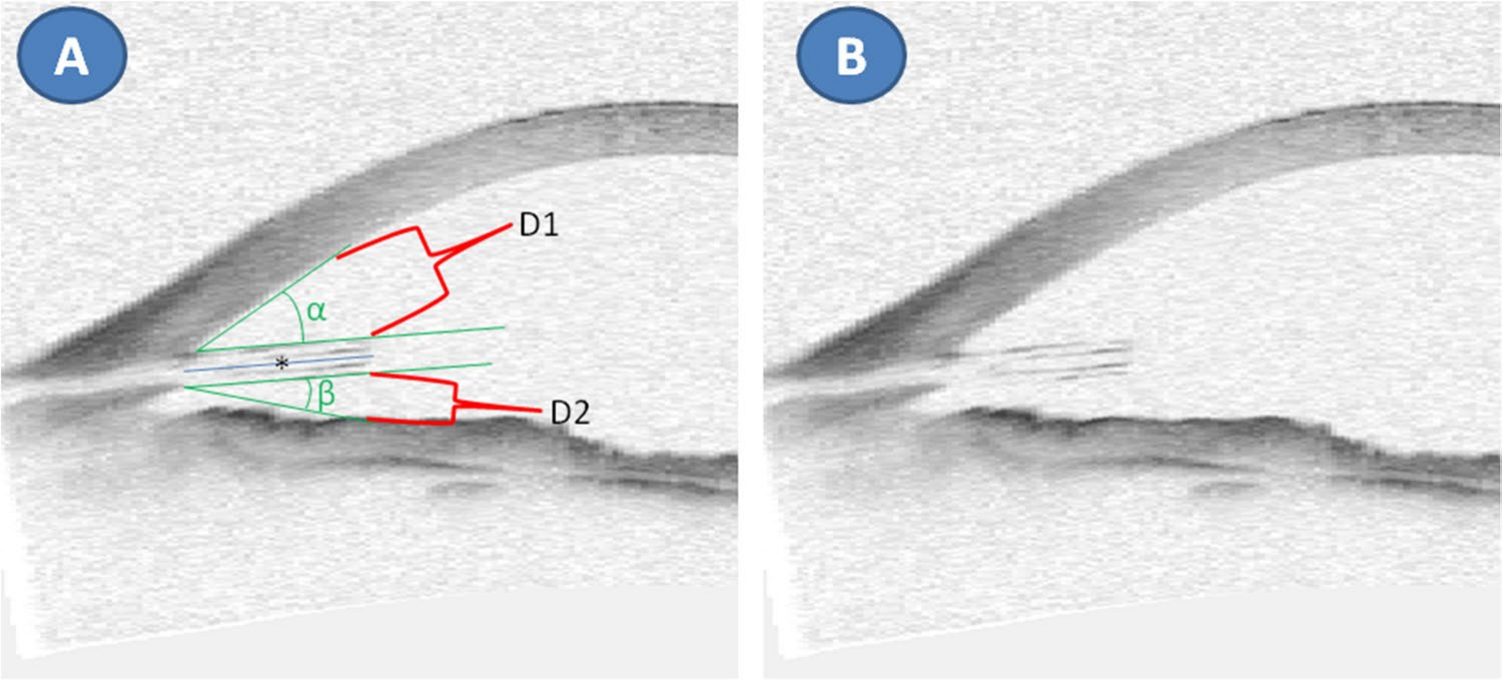
Parameters assessed by anterior segment OCT. Radial AS-OCT section showing the XEN45 stent with (**A**) and without (**B**) marked parameters in the anterior chamber: D1 = shortest distance outer diameter XEN tube to corneal endothelium; D2 = shortest distance outer diameter XEN tube to iris. Blue line with asterisk (*) shows the length of the XEN in the anterior chamber; α = angle XEN stent to the cornea; β = angle XEN stent to iris

Anterior segment optical coherence tomography (AS-OCT, Visante OCT, Zeiss; Fig. 2) was used to determine the position of the XEN45 in the anterior chamber. The distance of the XEN45 to the cornea, the angle between the tube and the cornea, the angle between the tube and the iris, and the tube length in the anterior chamber were recorded using anterior segment optical coherence tomography.

Using a Konan CellChek XL Specular Microscope (Konan Medical), endothelial cell densities were measured at 3 positions: central, superior-nasal (location of XEN45 implantation), and inferior-temporal (opposite the XEN45 implant).

Central corneal thickness was measured in the center of the cornea (Pocket II Pachymeter, Quantel Medical, France).

The same devices were used pre- and postoperatively for measurements of endothelial cell density and central corneal thickness.

Slit lamp and dilated fundus examinations were performed per clinical routine.

### Statistical methods

Data were checked for correctness and reasonable conditions. Continuous variables were also tested by using the Kolmogorov–Smirnov test. Friedman ANOVA, Kruskal–Wallis ANOVA, Fisher’s exact, Pearson’s Chi-Square test and Wilcoxon’s matched pairs, independent and dependent *t*-tests, and Spearman correlations were used to analyze data. Whisker plots were used to illustrate means/medians. For medians, Hodge-Lehman confidence intervals were computed. Global tests (e.g., Friedman ANOVA) over the whole time range lacked a large number of missing data, resulting in reduced power of the global test, and therefore local pairwise tests were also included (Wilcoxon-matched pairs tests) to increase power. Data was pooled into data bins for the first, second, third, fourth, and fifth postoperative years. Changes in absolute endothelial cell counts in each patient were expressed in percentages. This approach takes into account the individual level of endothelial cell counts of each patient at baseline and allows computation of 95% confidence intervals. An a posteriori power analysis was done to estimate the effect, i.e., loss of endothelial cell counts from baseline to 5-year follow-up (detectable with high power at the given sample sizes). All reported tests were two-sided, comparisons of endothelial cells were one-sided and *p*-values < 0.05 were considered statistically significant. All statistical analyses were performed using STATISTICA 13 (Hill, T. & Lewicki, P. Statistics: Methods and Applications. StatSoft, Tulsa, OK), NCSS 10 (Statistical Software (2015). NCSS, LLC. Kaysville, Utah, USA), MATHEMATICA 12 (Wolfram Research, Inc., Champaign, IL 2018), and PASW 24 (IBM SPSS Statistics for Windows, Version 21.0., Armonk, NY).

## Results

One hundred fifty-five eyes (129 patients) completed the study. An overview of baseline characteristics and demographic data is given in Table 1. The mean overall last postoperative visit was 3.7 (range 1.6–6.7) years after surgeries. The surgical history of the study population at baseline is presented in Table 2. Mean baseline IOP (23.9 [SD, 8.3] 12.6 [2.8]) and number of IOP-lowering medications (2.9 [1.0] 1.4 [1.2]) decreased statistically significantly at 5 years (both *p* < 0.001).

**Table 1:** Baseline characteristics and demographic data

	Solo surgery group	Combined surgery group	Both groups
Patients	61 (47%)	68 (53%)	129 (100%)
Eyes	69 (44%)	86 (56%)	155 (100%)
Age at surgery	68.3 (12.7) ^1^	74.4 (6.7) ^1^	71.4 (10.5) ^1^
Age at visit	72.1 (12.6) ^1^	78.0 (6.9) ^1^	75.4 (10.3) ^1^
Interval between surgery and visit (mean years; range)	3.8 (1.6–6.7)	3.6 (1.6–6.7)	3.7 (1.6–6.7)
Male/female (%)	34/35 (49%/51%)	32/54 (37%/63%)	66/89 (43%/57%)
Right/left eye (%)	35/34 (51%/49%)	34/52 (40%/60%)	69/86 (45%/55%)
History of past cataract surgery (%)	22/69 (32%)*	0/86 (0%)	-
IOP (mmHg)	23.9 (9.04) ^1^	24.0 (7.73) ^1^	23.9 (8.28) ^1^
Number IOP-lowering medications	3.01 (0.96) ^1^	2.84 (0.93) ^1^	2.93 (0.96) ^1^
Endothelial cell count (central)	2534 (2375–2584)^2^	2532 (2457–2577)^2^	2538 (2463–2571) ^2^
Central corneal thickness (µm)	529 (43)^1^	522 (39) ^1^	525 (41) ^1^
Mean deviation in visual field (dB)	− 11.8 (9.45)^1^	− 12.5 (8.6) ^1^	− 12.2 (9.0) ^1^
Best-corrected visual acuity (logMAR)	− 0.72(1.39)^1^	− 0.64 (1.05)^1^	− 0.68(1.21)^1^

Solo surgery group: Solo XEN45 procedure; combined surgery group: combined surgery/cataract surgeries

^1^Mean (Std)

^2^Median with 95% CI; besides a history of cataract surgery, all baseline characteristics were not significantly statistically different between subgroups (all *p* > 0.05)

^*^13/47 (28%) had cataract surgery between the XEN45 solo procedure and postoperative visit, which took place an average of 3.8 years after the XEN45 solo procedure

**Table 2:** Surgical history at baseline

	Solo surgery group	Combined surgery group	Both groups
Eyes*	69	86	155
SLT	25 (36%)	24 (28%)	49 (32%)
Solo cataract surgery	22 (32%)	-	22 (14%)
YAG iridotomy	9 (13%)	0 (0%)	9 (6%)
YAG capsulotomy	1 (1%)	-	1 (1%)
AntiVEGF intravitreal injection	1 (1%)	1 (1%)	2 (1%)
XEN	0 (0%)	1 (1%)	1 (1%)
Trabeculectomy	0 (0%)	1 (1%)	1 (1%)
Needling	0 (0%)	1 (1%)	1 (1%)
Viscokanaloplasty	1 (1%)	0 (0%)	1 (1%)
Retinal laser	1 (1%)	1 (1%)	2 (1%)
None	30 (43%)	56 (65%)	86 (55%)

Solo surgery group: Solo XEN45 procedure; combined surgery group: combined surgery/cataract surgeries

^*^Eyes could have had multiple past surgeries at baseline. The surgical history did not statistically significantly influence the endothelial cell count at baseline (all *p* > 0.20)

### Endothelial cell count compared to baseline

**Fig. 3:**
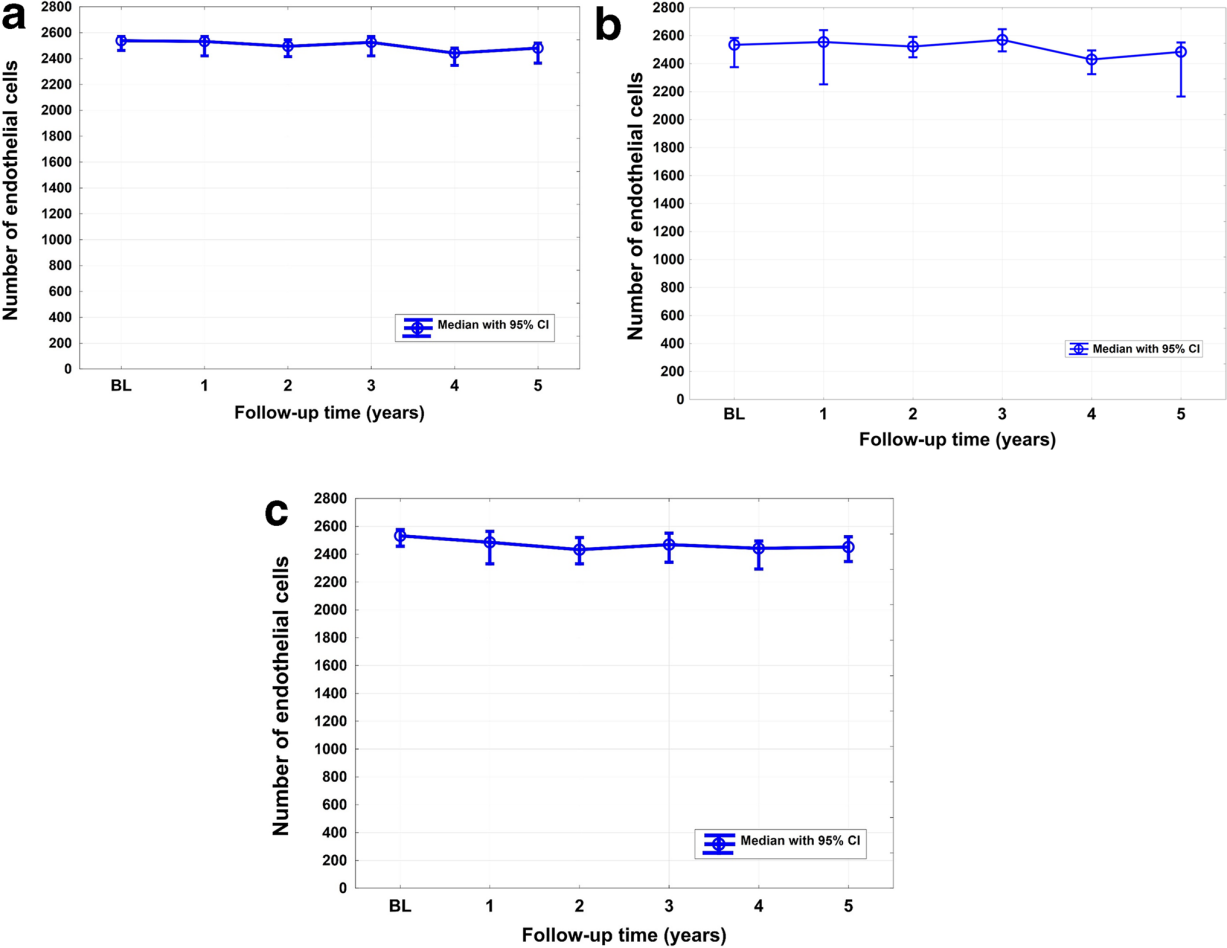
**A** Course of central endothelial cell count after XEN45 implantation in combination with or without cataract surgery over 5 years (overall). **B** Course of central endothelial cell count after XEN45 implantation without cataract surgery over 5 years (solo surgery group). **C** Course of central endothelial cell count after XEN45 implantation with cataract surgery over 5 years (combined surgery group)

Absolute means and medians and changes in endothelial cell counts expressed in percentages are displayed in Table 3. Results show that in some cases data deviated from normal or known distributions. Thus, parametric or semi-parametric models were not applicable and hypotheses were tested non-parametrically. Consequently, medians are used to report a change in endothelial cell count over time. Figure 3 A, B, and C illustrate the course of median endothelial cell count over time.

**Table 3:** Change in endothelial cell count over time

	Solo surgery group			Combined surgery group			Both groups		
	Median	Median % change^*^	Mean	Median	Median % change^*^	Mean	Median	Median % change^*^	Mean
Baseline	2534 (2375 to 2584, *n* = 69)	-	2439 (2350 to 2532, *n* = 69)	2532 (2457 to 2577, *n* = 86)	-	2511 (2457 to 2566, *n* = 86)	2538 (2463 to 2571, *n* = 155)	-	2482 (2432 to 2533, *n* = 155)
1 year	2555 (2252 to 2639, *n* = 16)	3% (− 3 to 18, *n* = 16)	2485 (2345 to 2634, *n* = 16)	2485 (2331 to 2523, *n* = 20)	2% (− 1 to 5, *n* = 20)	2448 (2379 to 2520, *n* = 20)	2532 (2421 to 2571, *n* = 36)	2% (− 1 to 5, *n* = 36)	2465 (2390 to 2541, *n* = 36)
2 years	2523 (2445 to 2591, *n* = 30)	0% (− 3 to 2, *n* = 30)	2498 (2409 to 2590, *n* = 30)	2433 (2331 to 2519, *n* = 40)	− 3% (− 7 to 0, *n* = 40)	2430 (2358 to 2504, *n* = 40)	2494 (2415 to 2545, *n* = 71)	− 1% (− 3 to 0, *n* = 71)	2459 (2402 to 2516, *n* = 71)
3 years	2571 (2488 to 2646, *n* = 31)	2% (− 2 to 4, *n* = 31)	2523 (2417 to 2634, *n* = 31)	2469 (2345 to 2593, *n* = 29)	0% (− 7 to 1, *n* = 29)	2434 (2352 to 2519, *n* = 29)	2525 (2421 to 2571, *n* = 61)	0% (− 3 to 2, *n* = 61)	2482 (2414 to 2552, *n* = 61)
4 years	2430 (2325 to 2494, *n* = 28)	− 1% (− 8 to 3, *n* = 28)	2306 (2178 to 2442, *n* = 28)	2442 (2292 to 2494, *n* = 32)	− 4% (− 9 to 1, *n* = 32)	2346 (2222 to 2465, *n* = 32)	2442 (2347 to 2481, *n* = 60)	− 3% (− 7 to 1, *n* = 60)	2327 (2241 to 2417, *n* = 60)
5 years	2485 (2165 to 2551, *n* = 18)	− 1% (− 14 to 8 *n* = 18)	2236 (2001 to 2500, *n* = 18)	2451 (2347 to 2525, *n* = 20)	− 1% (− 9 to 4, *n* = 20)	2354 (2210 to 2508, *n* = 20)	2481 (2364 to 2519, *n* = 39)	− 1% (− 7 to 4, *n* = 39)	2306 (2170 to 2450, *n* = 39)

Solo surgery group: Solo XEN45 procedure; combined surgery group: combined surgery/cataract surgeries

^*^Change in absolute endothelial cell count of each patient was expressed in percentages. Note that this approach takes individual endothelial cell count at baseline into account allowing for the computation of 95% confidence intervals. Results are given in terms of medians as well as means and 95% CI. Although testing of results was conducted non-parametrically due to deviated data distribution from normal or known distributions, we here show means additionally. This increases comparability with other published results

Compared to baseline endothelial cell count, no significant overall reduction could be shown at the last postoperative visit (*p* = 0.76). Pairwise tests show a significant reduction at years 2 and 4 (*p* = 0.005 and *p* = 0.035, both one-sided). The median reduction of endothelial cell count is − 30 (95% CI: − 85 to 0) and − 79 (95% CI: − 157 to 36) cells, respectively. Pairwise comparisons at years 1, 3, and 5 were not statistically significant (all *p* > 0.06).

When stratifying by type of surgery, the combined surgery group does not show a significant reduction when tested globally (*p* = 0.44). Again, we provide results of pairwise tests showing that a significant reduction was found at years 2 and 4 (*p* = 0.001 and *p* = 0.02, both one-sided). The median reduction of endothelial cell counts are − 79 (95% CI: − 183 to − 9) and − 93 (95% CI: − 220 to 23) cells, respectively. Pairwise comparisons at years 1, 3, and 5 are not statistically significant (all *p* > 0.09).

The solo surgery group does not show a significant reduction when tested globally (*p* = 0.16). Pairwise tests do not show any significant reduction at the last postoperative visits (all *p* > 0.07).

A posteriori power analysis reveals that our study would detect an average loss of − 130 endothelial cells from baseline to 5-year follow-up (corresponding to − 5.4%) with a power of 87% and a sample size of 39. We can thus conclude that it is highly likely that the average overall loss is less than − 130 endothelial cells at 5 years. In fact, taking into consideration the full data of all 39 patients at baseline and 5-year follow-up, the observed average loss is − 86 endothelial cells overall.

The proportion of eyes with a loss of > 20% and > 30% is given in Table 4.

**Table 4:** Proportion of eyes with an endothelial cell count loss of > 20% and > 30% after XEN45 surgery with and without combined cataract surgery at visit compared to baseline (%)

Time of assessment	Loss > 20%/loss > 30%		
	Solo surgery group	Combined surgery group	Both groups
1 year	0%/0% (*n* = 16)	5%/0% (*n* = 20)	3%/0% (*n* = 36)
2 years	7%/3% (*n* = 31)	3%/0% (*n* = 40)	4%/1% (*n* = 71)
3 years	0%/0% (*n* = 32)	3%/0% (*n* = 29)	2%/0% (*n* = 61)
4 years	4%/4% (*n* = 28)	13%/9% (*n* = 32)	8%/7% (*n* = 60)
5 years	11%/6% (*n* = 19)	5%/5% (*n* = 20)	8%/5% (*n* = 39)

Solo surgery group: Solo XEN45 procedure; combined surgery group: combined surgery/cataract surgeries

### Endothelial cell count compared under postoperative visits

Pairwise comparison of absolute loss of median endothelial cell count over time reveals a significant decrease from year 1 to year 4 (− 81, 95% CI: − 155 to − 8, *p* = 0.017, one-sided, *n* = 23) and year 2 to year 3 (− 83, 95% CI: − 186 to + 4, *p* = 0.035, one-sided, *n* = 20).

### XEN45 location parameters and association with endothelial cell count change

Analysis of the XEN45 implant location in the angle visualized by gonioscopy shows that 121/155 (78%) of the eyes had their final implantation in non-pigmented and pigmented trabecular meshwork. All other locations showed a rate between 1 and 5%.

Explorative data analyses reveal no statistically significant difference related to a higher loss of central endothelial cells in the following parameters:

Length of XEN45 in the anterior chamber (*p* = 0.09), angles of XEN45 to the cornea (*p* = 0.33) and iris (*p* = 0.80) in OCT, distances between inner ostium of the implant and cornea (*p* = 0.40) or iris (*p* = 0.09) in OCT, location of implantation in the angle visualized by gonioscopy (*p* = 0.81), XEN45 lumen endothelial touch in gonioscopy (*p* = 1.00).

### Further results at the last postoperative visit

**Fig. 4:**
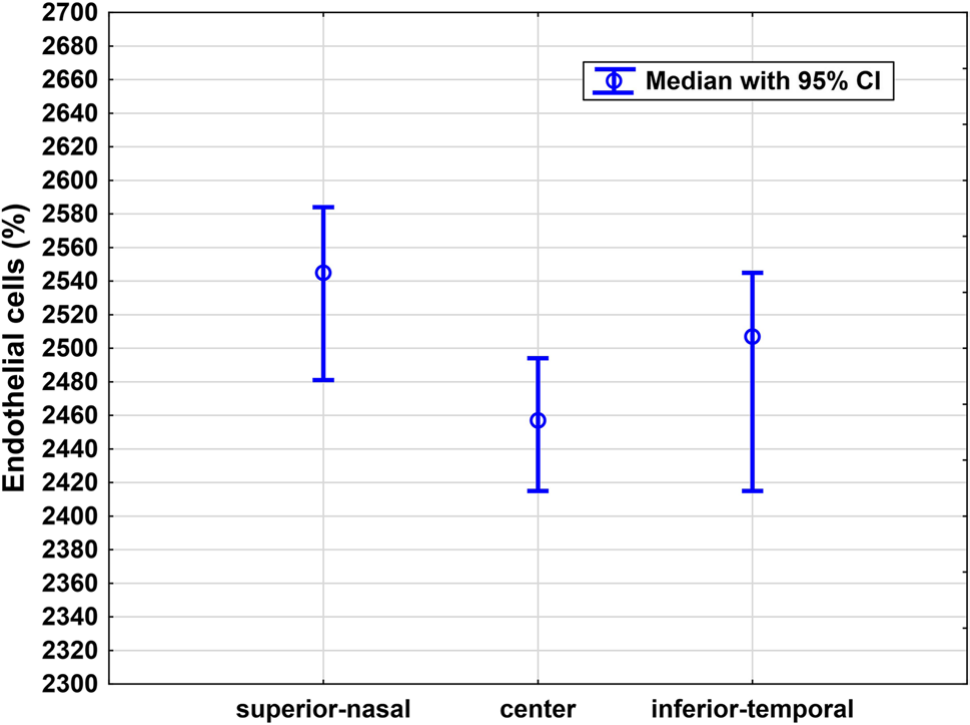
Endothelial cell counts at different locations measured at the last postoperative visit. There is no statistically significant difference between central, superior-nasal, and inferior-temporal endothelial cell count measurement (Friedman ANOVA, *p* = 0.1)

At the last postoperative visit, median central endothelial cell count (2457 [95% CI: 2415–2494]) highly correlates with the superior-nasal (2545 [2481–2584], *r* = 0.75) as well as inferior-temporal (2519 [2415–2545], *r* = 0.76) endothelial cell count (both *p* < 0.001). There is no statistically significant difference between central, superior-nasal, and inferior-temporal endothelial cell count measurement (*p* = 0.1, Fig. 4).

### Mean central corneal thickness

In the overall group, the mean central corneal thickness significantly reduces from 523.3 (SD ± 40.9) at baseline to 518.3 (± 36.4) at the last postoperative visit (*p* = 0.007). In the combined surgery group mean CCT shows a significant statistical reduction from 522.1 (± 38.9) to 515.3 (± 32.5, *p* = 0.0017). No loss in mean CCT was found in the solo surgery group (*p* = 0.18).

### Secondary surgeries

We analyzed the influence of secondary surgeries (needling, bleb revisions, laser iridoplasty, or YAG lysis of XEN45 at inner lumen due to suspected occlusion) on change in central endothelial cell count. No significant factor influencing the change of central endothelial cell count at any follow-up time was found (all *p* > 0.05).

## Discussion

Corneal endothelial cell loss is a well-described complication after anterior segment surgery and has been reported after filtering and non-filtering glaucoma surgeries [18–21]. Even after standard trabeculectomy with or without mitomycin C, endothelial cell loss of − 9.6 to − 10% have been reported in three different studies at 12 months [18, 22, 23]. The CyPass implant (Alcon Inc., USA) in combination with cataract surgery showed a decrease of − 20.4% in endothelial cell count at 5 years [24]. As a consequence, there was a concern that endothelial cell loss might be a caveat with the use of other implants, e.g., the XEN45 gel stent. Hence, the present study sought to investigate endothelial cell count loss after XEN45 implantation with or without combined cataract surgery during a follow-up of 5 years after surgery.

In both the solo surgery group as well as in the combined surgery group, the observed loss of endothelial cell count was − 1% (95% CI: − 7 to 4%) at 5 years compared to baseline. In general, the observed reduction of endothelial cell count was less or equal to − 4% after 1–5 years of follow-up in the entire study population as well as in the solo and the combined surgery subgroups.

Pairwise comparisons of baseline and postoperative visits revealed a decrease in central endothelial cell counts at 2 and 4 years postoperative overall (− 1%, − 3%) as well as in the combined surgery group (− 3%, − 4%). No statistically significant change was found for 1, 3, and 5 years postoperatively. Our data further suggest a slow, continual decrease of endothelial cell counts over time after combined surgery/cataract surgeries as opposed to a single decrease in endothelial cell count due to surgical trauma without a further decrease of endothelial cell counts over time. We want to point out that solo cataract surgeries can lead to a decrease in central endothelial cell counts (up to − 21% after 11 years) [25, 26]. In the combined surgery cataract group, the median reduction was − 79 (95% CI: − 183 to − 9) at 2 years and − 93 (95% CI: − 220 to − 23) cells at 4 years, corresponding to a mean reduction of − 3% (− 7 to 0) at 2 years and − 4% (− 9 to − 1) at 4 years. Taking into consideration the long follow-up in this study, this seems to be of low clinical relevance, since most XEN45 patients have > 2000 cells preoperatively.

The observed differences between solo and combined surgery groups in the study could be explained by the surgical step of removal of the lens and implanting an IOL, which could put more stress on the endothelial cells in the combined surgery group.

The solo surgery group showed no statistically significant decrease in central endothelial cell counts in all postoperative visits (1–5 years).

### Comparison of our data with the XEN45 literature

Olgun et al. reported a loss of − 2.1% (reduction of means from 2156 to 2098, 49 eyes) 3 months after solo surgery implantation [18]. We could not show any significant change in the combined or solo surgery group in our study within the first years postoperatively. The longest retrospective observation period in the literature is provided by Gillman and coworkers who report corneal endothelial cell density over 2 years after combined surgery cataract implantation [27] in which the authors included 17 eyes in their analysis. Gillmann et al. report a decrease of − 14.3% (− 339 cells) at 2 years [27]. There was no statistically significant difference to the solo cataract control group in their study, and whether or not needling revisions were necessary did not influence the decrease of endothelial cells in their study [27]. Our results suggest a smaller decrease of central endothelial cell count over 5 years since we show a decrease of fewer than 130 cells (and less than − 5.4%) at 5 years. The surgical implantation technique was the same in our and their study: especially mitomycin C dosage, amount and route of application, ports setup, and the use of gonioscopy during XEN45 implantation were the same as in their study. However, Gillmann et al. presented no information about the cataract part of the combined surgery. It is relevant to point out that we use a higher frequency of corticosteroid drops in the first week and we prescribed preservative-free eye drops postoperatively. Needling revisions were performed without the use of mitomycin C. Furthermore, the proportion of patients in our study with a loss of more than 20% central endothelial cells postoperatively was lower in comparison to the study by Gillmann et al. They reported a loss of ≥ 20% at 2 years in 3/17 (18%) of the eyes after combined surgery cataract implantation while we detected a decrease of > 20% in 3% of the eyes at 2 years [27].

### XEN45 location parameters and association with endothelial cell count change

The distance of the Ahmed tube tip to the cornea has a significant association with endothelial cell loss in the quadrant of implantation [13–15]. Tubes that are closer to the cornea seem to lead to increased loss of adjacent endothelial cells (2.5 years after implantation) [12].

Surprisingly, the placement of the XEN45 implant in the anterior chamber angle (e.g., more anterior near the Schwalbe line, more posterior near the scleral spur) and the distance of inner ostium to cornea had no statistically significant influence on the course of central endothelial cell count after XEN45 implantation.

Further to this, after Ahmed valve implantation, the lowest endothelial cell count was recorded at the site of implantation, suggesting that the silicone tube of the Ahmed valve locally reduces endothelial cell counts [28–30]. The data of the present study do not support a similar local effect at the entry site of the XEN45 implant. Comparing the endothelial cell count at the implant (measurement superior-nasal) and at the opposite side of the cornea (measurement inferior-temporal) showed a homogenous distribution of endothelial cells over the cornea.

Therefore, the tolerance zone of implantation seems to be large with regard to central endothelial cell loss, possibly giving the surgeon more freedom in terms of positioning the implant at the chamber angle. Despite these findings, we recommend being cautious when choosing the site of implantation, i.e., not implanting too anterior or posterior, as placement could influence other factors (e.g., efficacy and safety: IOP outcome, revision rates, re-surgery rates, needling rates, and the rate of adverse events).

### Possible mechanism(s) of loss of central endothelial cell density after glaucoma surgery

The exact mechanism of corneal endothelial damage is unclear [18]. In the case of the silicon tube valve, some authors postulate that jet flow through the silicone tube is caused by the heartbeat, chronic chamber inflammation, mechanical loss by intermittent tube-endothelial cell contact when the patient blinks or rubs the eye, tube-uveal touch, and a foreign body reaction to the silicone might play a role. Further turbulence present at the tip of the implant or microscopic fibrotic tissue between the tube and corneal endothelium may be a possible mechanism for the decrease of endothelial cell count after Ahmed valve implantation [12, 31–33]. These reasons might also be causative for a decrease in endothelial cell count after XEN45 implantation. Compared to the low-resistance silicone tubes of plate implants, the XEN45 has a smaller outer diameter, a smaller length in the anterior chamber, and a lower maximal flow rate due to the smaller lumen size, and is made of a less rigid material [18]. This is speculation of course; however, it is not without reason to assume that these factors might account for a beneficial outcome in the endothelial cell count data postoperatively with XEN45 compared to big plate glaucoma devices, which were shown to cause an endothelial cell count loss of − 11.5 to − 18.6% at 24 to 36 months [13–15, 34]. Postoperative interventions (e.g., needlings, bleb revisions) do not seem to have any apparent influence on endothelial cell loss after XEN45 implantation.

Whether normal aging, the cataract part of the combined surgery cataract surgery, the XEN45 per se, or a combination of these factors is the reason for the slow decrease in this group, cannot be distinguished with our data. Further investigations and analyses are suggested.

### Strengths of the study

To date, a 2-year retrospective follow-up is the longest published dataset. There is no mid-term data on solo surgery procedures published yet. With this study, we investigated the mid-term course of the endothelial cell count on solo surgery procedures.

### Limitations of the study

Our study population was strictly Caucasian; therefore, caution is advised in generalization to other ethnicities.

Sample sizes decreased considerably over time. We therefore made an a posteriori power analysis revealing that a decrease in central endothelial cell counts of − 5.4% or more would have been detected by our study design with a power of 87% at the 5-year follow-up. This desirable result achieving high power is mainly due to the fact that endothelial cell counts are highly correlated (*r* = 0.65 between baseline and 5-year follow-up).

Attrition and loss of follow-up are important issues. However, after checking patients’ records, no evidence for systematic biases (e.g., patients with a lower or higher risk of loss of ECC due to pre-existing conditions or other reasons) was found which may distort study findings. A negative effect of a large loss of follow-up data is that 95% confidence intervals become wider.

No XEN45 location parameter is associated with a higher endothelial cell count loss. Any small clinical effect of XEN45 location parameters on postoperative endothelial cell count course cannot be answered with our data. Therefore, we would suggest follow-up studies with higher sample sizes and further increasing follow-up time.

## Footnotes

1 Three times a day.

2 Hourly.
